# Characterization and Hemocompatibility of α, β, and γ Cyclodextrin-Modified Magnetic Nano-Adsorbents

**DOI:** 10.3390/ijms251910710

**Published:** 2024-10-04

**Authors:** Mehdi Ghaffari Sharaf, Shuhui Li, Elyn M. Rowe, Dana V. Devine, Larry D. Unsworth

**Affiliations:** 1 Department of Chemical and Materials Engineering, University of Alberta, Edmonton, AB T6G 1H9, Canada; mghaffar@ualberta.ca (M.G.S.); shuhui1@ualberta.ca (S.L.); 2 Department of Pathology and Laboratory Medicine, University of British Columbia, Vancouver, BC V6T 1Z7, Canada; elyn.rowe@ubc.ca (E.M.R.); dana.devine@blood.ca (D.V.D.)

**Keywords:** chronic kidney disease, uremic toxin, adsorbent, cyclodextrin, magnetic nanoparticle, protein adsorption, hemocompatibility

## Abstract

Kidney dysfunction leads to the retention of metabolites within the blood that are not effectively cleared with conventional hemodialysis. Magnetic nanoparticle (MNP)-based absorbents have inherent properties that make them amenable to capturing toxins in the blood, notably a large surface area that can be chemically modified to enhance toxin capture and the ability to be easily collected from the blood using an external magnetic field. Cyclodextrins (CDs) present a chemical structure that facilitates the binding of small molecules. However, the hemocompatibility of MNPs modified with films composed of different native types of CDs (α, β, or γ) has not yet been investigated, which is information crucial to the potential clinical application of MNPs to supplement hemodialysis. To this end, films of α-, β-, or γ-CDs were formed on MNPs and characterized. The impact of these films on the adsorbed protein structure, composition of key adsorbed proteins, and clotting kinetics were evaluated. It was found that modified MNPs did not significantly affect the secondary structure of some proteins (albumin, lysozyme, α-lactalbumin). The adsorbed proteome from platelet-poor human plasma was evaluated as a function of film properties. Compared to non-modified nanoparticles, CD-modified MNPs exhibited a significant decrease in the adsorbed protein per surface area of MNPs. The immunoblot results showed variations in the adsorption levels of C3, fibrinogen, antithrombin, Factor XI, and plasminogen across CD-modified MNPs. The hemocompatibility experiments showed that CD-modified MNPs are compatible with human whole blood, with no significant impact on platelet activation, hemolysis, or hemostasis.

## 1. Introduction

Kidney disease is a global health issue that impacts more than 800 million people worldwide and contributes to global mortality, with a significant rise in associated fatalities over the last two decades [1]. A progressive and irreversible deterioration in kidney function leads to the build-up of metabolites—including biologically active uremic toxins—in the blood. Uremic toxicity, the result of accumulated uremic toxins, contributes to increased mortality and reduced quality of life in those with chronic kidney disease via damage to several organ systems [2]. Conventional, membrane-based hemodialysis targets the removal of low molecular weight compounds, including some uremic toxins, but is inefficient in eliminating those strongly bound by proteins. These protein-bound uremic toxins significantly contribute to the pathophysiology of kidney disease and other co-morbidities [1,3]. Adsorbent surfaces are thought to be a means to clear uremic toxins from the blood, which may also lead to wearable devices that potentially improve patient quality of life and treatment efficacy [4,5].

Cyclodextrins (CDs) have a hydrophilic outer surface and a relatively hydrophobic inner pocket that facilitates the binding of a diverse range of compounds. The difference in cavity volumes between the three native types of CDs (α-CD, 174 Å^3^; β-CD, 262 Å^3^; γ-CD, 427 Å^3^) may affect both the binding capacity and profile of adsorbed uremic toxins, and the hemocompatibility of the materials [6,7,8]. Additionally, some CDs have been shown not to trigger an immune response on their own [9]. Magnetic nanoparticles (MNPs) are widely used in biomedical applications due to their relatively good biocompatibility, maneuverability induced by external magnetic fields, and ease of surface modification [10]. MNPs coated with β-CD (β-MNPs) were used as absorbents for removing diazepam from blood [11]. MNP recovery was accomplished using an external magnetic field, while the β-CD film improved the overall biocompatibility and hemocompatibility [11]. Studies have demonstrated that β-MNPs do not exhibit any significant toxicity at the cellular level [12]. In addition to whole blood component interactions, understanding how CD-modified MNPs interact with blood proteins is crucial, as nanoparticles can induce conformational changes in protein structures, exposing occult epitopes and potentially triggering biological signals. Moreover, non-specific protein adsorption at the blood–material interface can initiate deleterious host responses, including activating the immune response and coagulation cascade [13]. The formation of blood clots involves a complex series of interconnected events, including the absorption of proteins, the enzymatic conversion of proteins for coagulation and complement system activation, and the adhesion and activation of platelets [13,14,15,16]. The effect that CD-modified MNPs have on overall protein–surface interactions, enzymatic action leading to the activation of coagulation and complement cascades, and platelet activation has yet to be delineated.

**Scheme 1:**
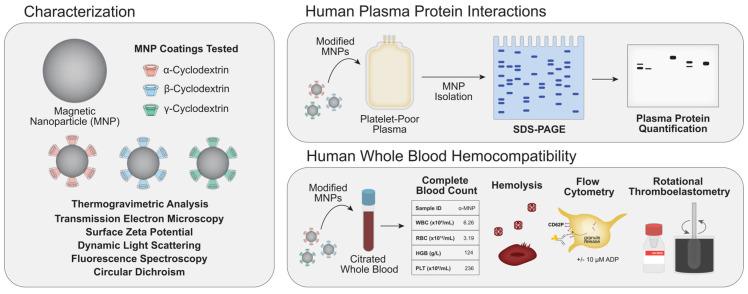
A schematic representation of the study. MNPs were functionalized with different types of CDs, including α-, β-, and γ-CD. A range of characterization techniques was employed, including TEM, DLS, TGA, and surface zeta potential measurements. Fluorescence spectroscopy and circular dichroism were utilized to study the interactions between proteins and the modified surfaces. The binding of human plasma proteins to CD-coated MNPs was evaluated, and hemocompatibility with human whole blood was assessed.

Applying CD-modified MNPs for the adsorption of uremic toxins in the blood necessitates an understanding of the effect their physicochemical properties may have on blood cells and hemostasis. To this end, CD films were formed on MNPs (Fe_3_O_4_) via co-precipitation and characterized using thermogravimetric analysis (TGA), transmission electron microscopy (TEM), dynamic light scattering (DLS), and surface zeta potential measurements. The adsorbed protein secondary structure as a function of the CD type was assessed using circular dichroism for human serum albumin (HSA), α-lactalbumin, and lysozyme (Scheme 1), where HSA and α-lactalbumin have a similar charge but differ in size to provide insights into size-related effects. Also, lysozyme and α-lactalbumin have a similar size but different charge [17]. Additionally, the adsorption-induced changes in HSA structure was studied by monitoring the changes in tryptophan fluorescence in the presence of MNPs [18]. The total adsorbed amount of plasma proteins and adsorbed proteins to MNPs from platelet-poor plasma were evaluated using the BCA assay and immunoblots. To evaluate platelet function and the general hemocompatibility of all MNP systems, MNPs were incubated with whole blood and platelet activation and responsiveness (flow cytometry), clot formation (rotational thromoboelastometry), hemolysis (spectrophotometry), and complete blood counts were determined as a function of coating type. Through a deeper understanding of the interaction between plasma proteins, blood cells, and MNPs coated with α-, β-, and γ-CD, it is possible to evaluate their hemocompatibility and potential for adsorbing toxins from the blood.

## 2. Results and Discussion

### 2.1. Magnetic Nanoparticle Properties

**Figure 1:**
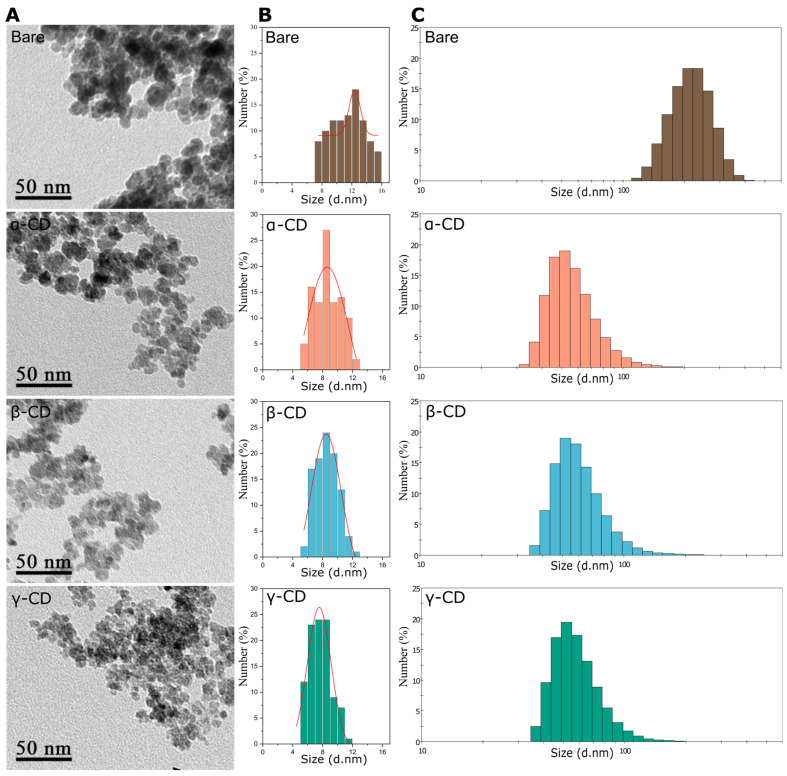
Representative MNP properties as characterized using TEM and DLS. (**A**) TEM micrographs of bare and coated MNPs. (**B**) Histogram representing the distribution of individual particle sizes obtained from TEM micrographs (n = 100 particles). (**C**) The size distribution of magnetic particle clusters was determined using DLS measurements (average diameter, n = 3).

In solution, MNPs are prone to aggregation, which has been previously shown to be influenced by size, charge, and morphology [19]. Using DLS, it was observed that bare, α-, β-, and γ-MNPs decreased in diameter from ~301 nm (PI: ~23) to ~94 nm (PI: ~0.14), 99 nm (PI: ~0.18), and 90 nm (PI: 0.19), respectively (Figure 1, Table 1). As observed previously, bare MNPs can form aggregates of 200–340 nm in diameter, and CD coatings inhibit MNP aggregation to form smaller more uniform clusters (ranging from ~94 to 170 nm in diameter) than bare MNPs [20,21]. It has previously been shown that coating MNPs with β-CD leads to the formation of smaller particles (dia. ~7.8 nm in diameter) compared to bare particles (~9.2 nm in diameter) [21]. Here, TEM micrographs showed a trend toward reduced particle sizes upon CD coating. In the case of bare particles, the grain size ranged from ~7 to ~15 nm. Conversely, CD-coated particles slightly shifted towards smaller grain sizes, ranging between ~6 and ~12 nm (Figure 1, Table 1). These findings support the previously observed role of CDs in augmenting particle properties for biomedical applications, which, in conjunction with citrate anions, reduce particle size and improve the uniformity of particles [21].

The zeta potential for bare MNPs was 5 ± 0.2 mV, a value commonly associated with not being colloidally stable [20]. The modified MNPs had negative zeta potentials: α-MNP, −24 ± 1.9 mV; β-MNP, −22 ± 0.6 mV; and γ-MNP, −22 ± 0.5 mV (Table 1). This trend towards significantly negative zeta potential values has been previously observed, where the zeta potential of bare MNPs measured −2.82 mV, while citrate-coated β-MNPs exhibited a value of −33.8 mV [21]. Moreover, the modified MNP zeta potentials likely underscore why these MNPs showed a lower degree of aggregation [22].

### 2.2. Film Hydration and Weight Loss

**Figure 2:**
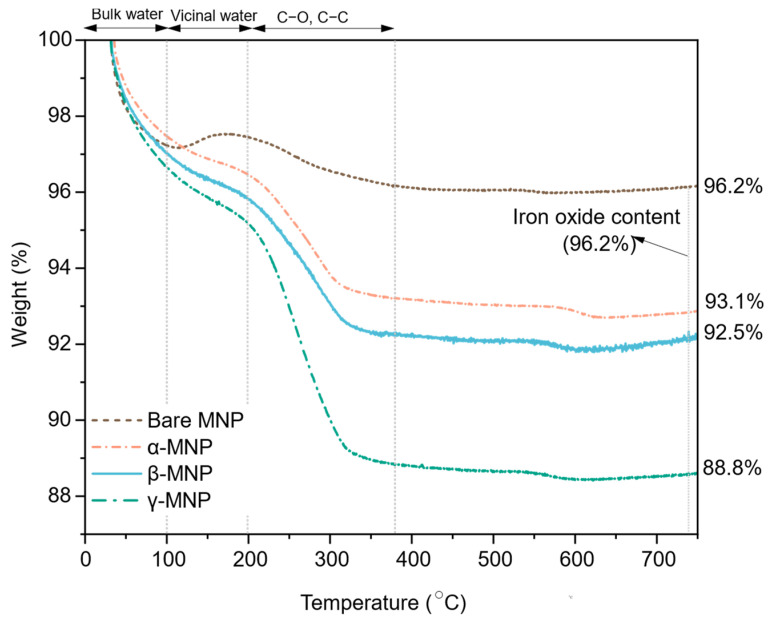
Representative thermograms of bare and CD-modified MNPs. The mass loss of the bare MNPs was 3.8 wt.%. In contrast, the α-, β-, and γ-MNPs had total mass losses of 6.9, 7.5, and 11.2 wt.%, respectively.

The CD film was further characterized using TGA, where a mass reduction observed for temperatures: (i) less than 100 °C was attributed to the release of bulk water; (ii) between 100 and 200 °C from the loss of vicinal water; and (iii) between 200 and 380 °C from the cleavage of C−O and C−C bonds. All modified MNPs systems showed a greater total mass loss than the bare control due to the thermal decomposition of the CD film: bare (3.8 wt.%) < α-CD (6.9 wt.% total, 3.1 wt.% due to CD) < β-CD (7.5 wt.% total, 3.7 wt.% due to CD) < γ-CD (11.2 wt.% total, 7.4 wt.% due to CD). This increasing trend corresponds to the increased number of glucose subunits, with α having six, β having seven, and γ having eight (Figure 2). CDs feature primary and secondary hydroxyl groups. These hydroxyl groups have the capacity to form hydrogen bonds with water molecules and other polar compounds. The hydrogen bonding capability increases from α- to γ-CDs due to the rising number of hydrogen bond donors and acceptors. The hydrogen bonding capacity is determined by the octanol/water partition coefficient (LogP_O/W_), where an increase in the number of hydrogen bond donors and acceptors correlates with a more negative LogP value. This trend has been observed in native CDs, with α-CD displaying a logP value of −12.7, β-CD at −14.82, and γ-CD at −16.93 [23]. Additionally, water molecules are present within the CD cavity, which can be displaced by hydrophobic guest molecules. Consequently, the variation in hydration level could be linked to the difference in cavity size, which also increases from α- to γ-CDs [24].

### 2.3. Effect of Coating on Adsorbed Protein Secondary Structure

**Figure 3:**
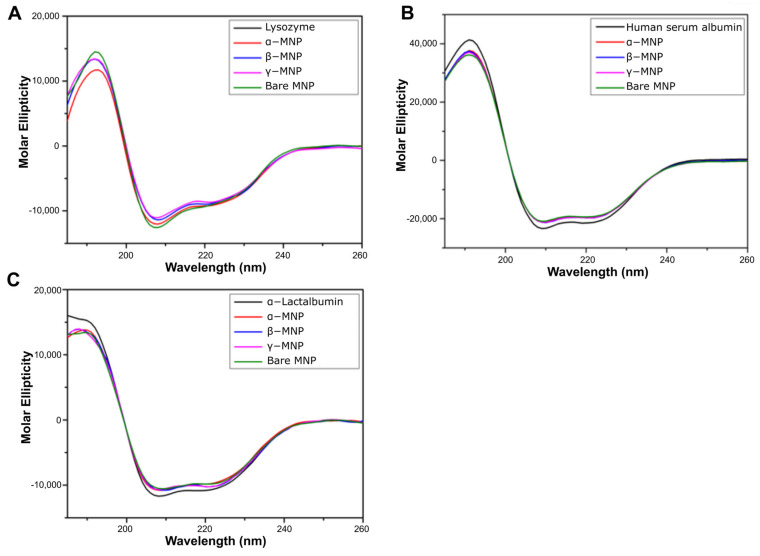
Circular dichroism spectra of protein-MNP. (**A**) Lysozyme, (**B**) HSA, and (**C**) α-lactalbumin in the presence of bare, α-, β-, and γ-MNP. All measurements are repeated three times.

The influence of surface chemistry for α-, β-, and γ-MNPs and bare MNPs on the structural characteristics of a subset of adsorbed proteins (i.e., lysozyme, HSA, or α-lactalbumin) was assessed (Table 2). Modified MNPs showed minimal impact on the secondary structure of HSA and α-lactalbumin (Figure 3B,C). However, in the case of lysozyme, a reduction of 2.1~2.3% in helix content and an increase of 0.1~0.4% in β-sheet content was observed compared to the native protein reference (Figure 3A). When examining HSA, both the modified and bare MNPs caused slight changes of less than 1% in the secondary structure of the protein. The α- and β-MNPs led to a slight increase in helix content by 0.1~0.3%, while the γ-MNPs resulted in a decrease of 0.6% in helix content. The γ-MNP surfaces induced a 0.6% increase in random coil content, whereas other engineered surface modifications caused less than 0.1% change in random coil content (Figure 3B). The conformational changes induced by the coated surfaces in α-lactalbumin were less than 1% compared to the control. Notably, γ-MNPs induced the most significant conformational changes in α-lactalbumin, reducing the helix content by 0.8% (Figure 3C). Previous studies using HSA incubated only with α-, β-, and γ-CD molecules have demonstrated that CDs do not penetrate the protein structure but rather interact with the protein’s surface. Importantly, this interaction does not noticeably alter the protein’s secondary structure, as determined using circular dichroism [25].

### 2.4. Effect of Coating on Adsorbed HSA Tertiary Structure

**Figure 4:**
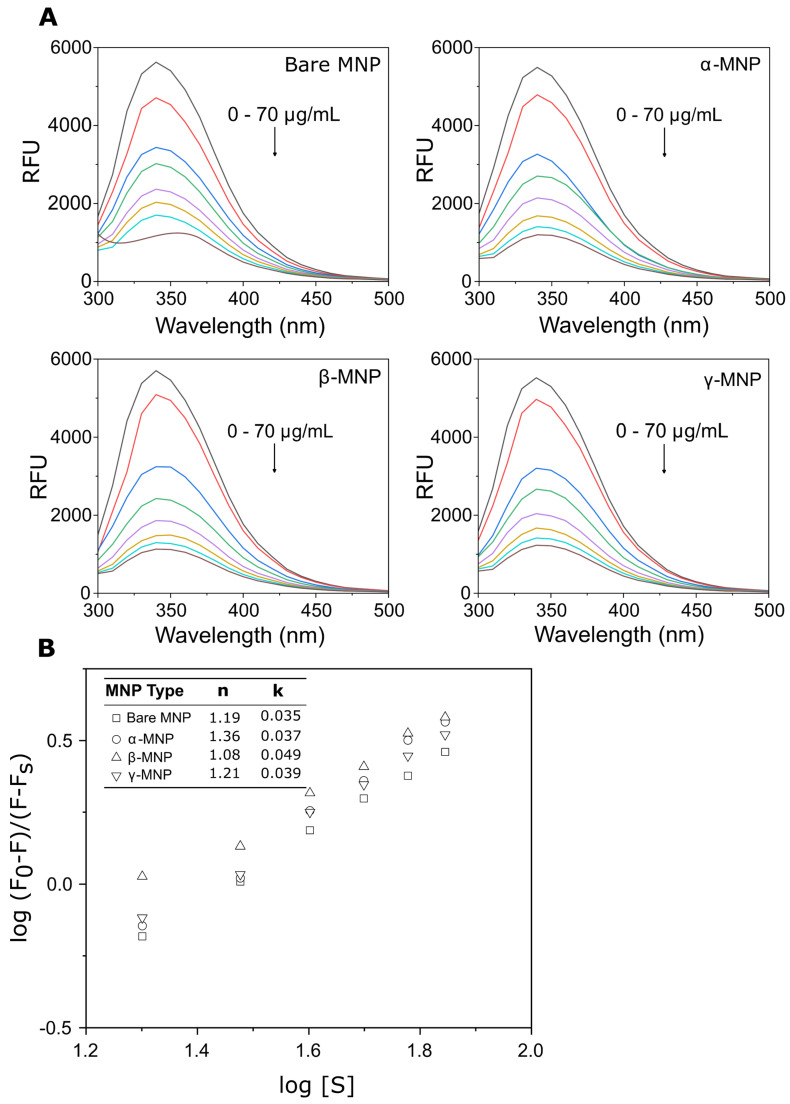
The interaction of HSA with bare and modified MNPs was investigated by quenching the intrinsic fluorescence of HSA in the presence of varied concentrations of nanoparticles. (**A**) Fluorescence emission spectra were generated by titrating the HSA solution with increasing concentrations of MNPs (0, 10, 20, 30, 40, 50, 60, and 70 μg/mL). (**B**) The plot yielded the number of binding sites (n) and the binding constant (K_a_), with data derived from the logarithmic relationship log [(F_0_ − F)/(F − F_s_)] vs. log [S]. Here, RFU stands for relative fluorescence units, [S] represents the concentration of MNPs, F_0_ corresponds to the relative fluorescence intensity (F) of the protein solution without MNPs, and F_s_ indicates the relative fluorescence intensity of the protein when fully saturated with MNPs. Data represent mean ± 1 SD, n ≥ 3.

Tertiary structural changes in HSA result in changes to the tryptophan microenvironment that alter its fluorescence [26]. Previous work has shown that the intrinsic fluorescence intensity of the tryptophan residue in HSA is reduced upon adsorption to MNPs and that the extent of this change is likely dependent upon the surface chemistry of particles [20]. Using a similar experiment, the number of protein binding sites (n) and the binding constant (K_a_) for bare and modified MNPs was determined (Figure 4). Notably, β-MNPs exhibited the fewest (n = 1.08) and α-MNP showed the highest (n = 1.36) number of binding sites. Bare MNPs had a binding site count of n = 1.19, with a slight increase observed with γ-CD-coated ones, showing n = 1.21. No substantial variations were observed in binding affinity towards HSA: bare, α-, β-, and γ-MNPs had k_a_ values of 0.037, 0.049, and 0.039, respectively (Figure 4B).

### 2.5. Total Adsorbed Protein

**Figure 5:**
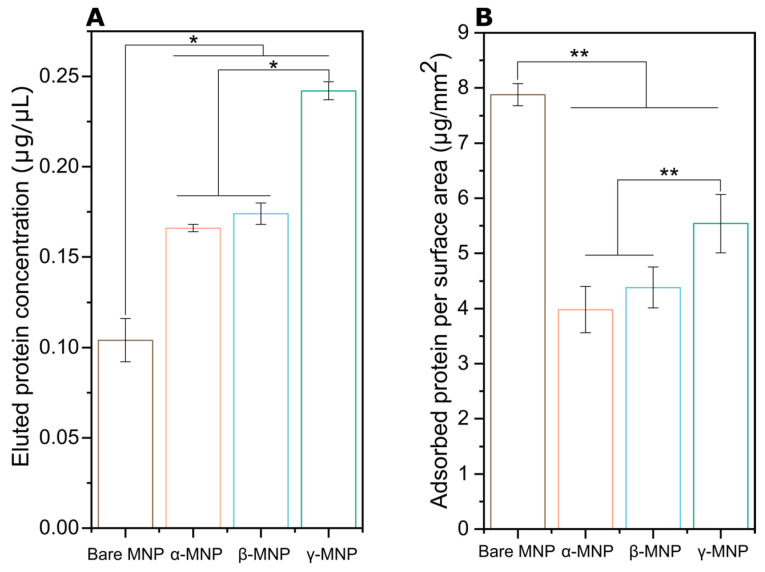
Representative BCA assay results illustrating adsorbed protein quantities. (**A**). Adsorbed protein concentration (μg/μL). (**B**). Adsorbed protein per surface area (μg/mm^2^). Statistical analysis was conducted using one-way ANOVA, followed by a post hoc Tukey’s HSD test. Where * and ** indicate statistical significance of *p* < 0.001 and *p* < 0.05, respectively. The data are the mean ± 1 SD, n ≥ 3.

Detergent-compatible BCA assay was employed to quantify the adsorbed amount of plasma proteins eluted from MNPs upon incubation in platelet-poor plasma (Figure 5). When considering the adsorption to these systems, the constant mass of MNPs was used to normalize the system. Without correcting for differences in surface area between samples, it was observed that more protein (*p* < 0.001) was eluted from modified versus bare MNPs: bare −0.10 µg/µL, α-MNP and β-MNP −0.17 µg/µL, and γ-MNP −0.24 µg/µL (Figure 5A). In prior studies on MNPs grafted with poly(β-CD), a notable decrease (*p* < 0.05) in plasma protein adsorption compared to bare MNPs was observed, where the plasma proteins eluted from bare and poly(β-CD)-grafted MNPs were measured at 0.3 µg/µL and 0.26 µg/µL, respectively [27]. When accounting for surface area differences between these systems, it was apparent that bare MNPs adsorbed more protein than the modified systems (Figure 5B). Given that α-, β- and γ-MNP have similar accessible surface area, it seems the coating properties affect protein adsorption. The difference in adsorbed protein may arise from the larger hydrophobic cavity in γ-CD compared to α- or β-CD and the difference in the number of glucose subunits [28].

### 2.6. Coating Effect on Coagulation

**Figure 6:**
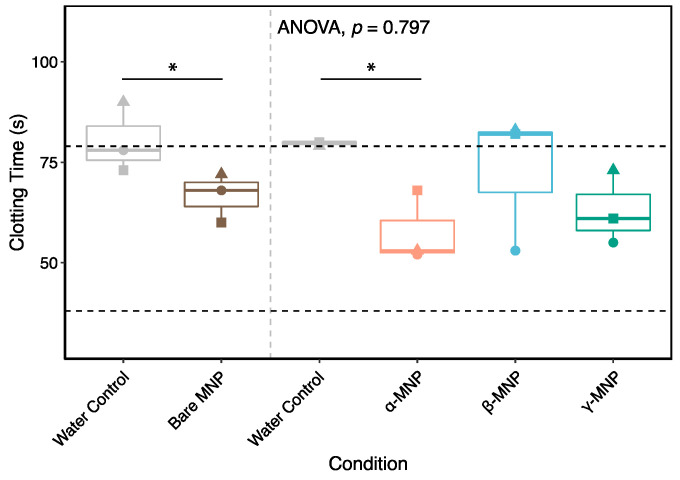
The presence of MNPs reduces clotting time driven by plasma proteins. Clotting time in whole blood as measured by rotational thromboelastometry (ROTEM) with extrinsic activation. Data on the left side of the dotted line represents controls conducted and published by our group [27]. Results were compared across groups with repeated measures ANOVA to compare differences within biological replicates across groups, and paired t-tests were used for pairwise comparisons to the water control (* *p* < 0.05). Comparisons not shown were not statistically significant.

Coagulation was assessed in whole blood through rotational thromboelastometry (ROTEM) using an extrinsic pathway clot initiator (EXTEM) (Figure 6). The results demonstrated a decrease in the clotting time for bare, α-, and γ-MNPs, indicating more rapid fibrin polymerization. Although not statistically significant due to the small sample size used for these preliminary hemocompatibility experiments, the trend for γ-MNP is clear, and it neared statistical significance when compared with the control (*p* = 0.097). However, β-MNP did not have this effect of reducing clotting time in two of the three donors in this set of experiments. The reason for this discrepancy in results in two of the donors is unclear. Previous work characterizing β-CD/MPC films on MNPs [27] showed a decrease in clotting time compared to control. That said, the β-CD/MPC films are formed via living polymerization and are very different in composition and structure than these physically anchored films.

### 2.7. Quantification of Plasma Proteins Adsorption

Immunoblot band intensities for plasma proteins eluted from all MNP systems were quantified using a 13-step grayscale system (Figure S1) for experiments that used consistent amounts of loaded protein and color development times (Table 3).

Albumin (66.5 kDa) is the most prevalent protein in plasma (35–50 g/L) and binds various metabolic compounds, lipids, and drugs [28]. Albumin adsorption can influence subsequent protein–surface interactions and coagulation processes [29,30]. In general, albumin has a higher affinity for hydrophobic surfaces, and as previously demonstrated, the incorporation of β-CD into hydroxyapatite nanoparticles has been shown to enhance albumin adsorption to that surface [31]. In this study, all MNPs, including bare, α-, β-, and γ-MNP, had a similar and relatively high amount (~9 scale) of eluted albumin. No trend in intensity was observed with CD modification. Previous immunoblot studies have demonstrated a slight reduction in albumin adsorption levels on β-CD-grafted MNPs in comparison to bare particles, albeit with relatively high intensity values observed for both unmodified and modified MNPs [27].

### 2.8. Immune Response-Related Proteins

The complement system is critical to the immune response to biomaterials, and C3 activation plays a central role in this response [32,33]. C3 can show for bands in the following immunoblots: full-length (187 kDa), α-chain (115 kDa), β-chain (70 kDa), and activation fragment (42 kDa) [34]. Herein, intact C3 was detected, with similar and moderately low intensities (~3–4 scale) compared to other proteins eluted for all MNPs. The α-chain intensities were lower between β- and γ-MNPs (3 scale) compared to α-MNPs and bare MNPs (~5–6 scale). The β-chain displayed relatively high band intensities across all types of MNPs. C3 activation fragments were found to be consistent for all systems (6 scale), indicating that CD modification did alter activation of C3 compared to controls. Comparable results have been reported for the intensity levels of β-chain and activation fragments in β-CD-grafted MNPs [27].

**Figure 7:**
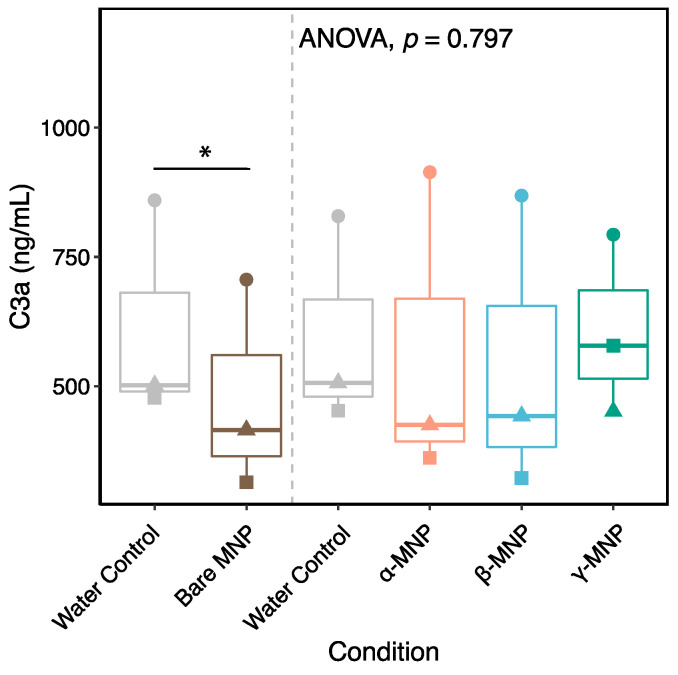
C3a ELISA results of MNP-depleted plasma from whole blood hemocompatibility experiments. Whole blood from n = 3 healthy donors was incubated with 0.18 mg/mL of each MNP formulation, and MNP-depleted plasma was assayed for C3a with a commercial ELISA. Data on the left side of the dotted line is from a separate set of experiments, previously published by our group [27], and shapes reflect biological replicates. Results were compared across groups with repeated measures ANOVA to compare differences within biological replicates across groups, and paired t-tests were used for pairwise comparisons to the water control (* *p* < 0.05). Comparisons not shown were not statistically significant.

ELISA was further used to quantify C3a in MNP-depleted frozen plasma from whole blood samples post-incubation (Figure 7). It was found that C3a concentrations were higher than expected in healthy donors [35]. This was likely a direct result of the ex vivo manipulation of the whole blood required for MNP treatment and subsequent manipulation of the plasma for MNP removal via centrifugation and magnet exposure, wherein a total of ~3 h elapsed between blood draw and the freezing of plasma for C3a analysis; the ex vivo complement activation continues in citrated plasma [32], which likely inflated the values reported here. Variability in responses across donors was observed; two of the donors showed decreased C3a levels relative to the control with the CD-MNPs. The only statistically significant difference was the reduction in C3a for bare MNPs compared to controls. Notably, the ELISA was performed in plasma depleted of MNPs following treatment, meaning the proteins bound to the MNPs (Table 3) were not measured. The fact that similar levels of C3a to the control are observed across all modified MNPs aligns with immunoblot results.

IgG is 10–20% of the total plasma protein complement and can initiate the classical complement pathway activation [34,36]. No change in band intensity was observed for IgG upon MNP modification. The heavy chain (55 kDa) showed lower intensity levels compared to the light chain (27 kDa). The presence of bound IgG may indicate a propensity for complement activation. Previous immunoblot studies have revealed a consistent trend in band intensity for the IgG light chain on β-CD-grafted MNPs, while a decrease in band intensity was observed for the IgG heavy chain upon β-CD grafting [27].

Factor I (88 kDa) regulates complement through cleaving complement proteins C3b and C4b [37]. Factor I was not observed for any MNP system. While IgG was present, the absence of Factor I may suggest that the classical activation pathways are not being regulated [38]. This finding is consistent with previous studies where Factor I has not been found adsorbed to β-CD-grafted MNPs [27]. Transferrin (77 kDa) can activate macrophages and function as an innate immune system component [32,39,40]. The adsorption of transferrin was consistent and relatively high across all types of MNPs, suggesting that the binding of this protein could potentially trigger macrophage activation. Similar levels of transferrin adsorption have been found in β-CD-grafted MNPs [27].

Vitronectin is a multifunctional glycoprotein that regulates the complement system in plasma [41] and preferentially interacts with negative or hydrophobic surfaces [42,43]. Similar and high-intensity bands for vitronectin were observed for all MNPs. Adsorbed fibronectin and vitronectin can enhance platelet adhesion, where fibronectin can also regulate platelet activity [44]. Despite the identical plasma concentrations of fibronectin and vitronectin, research on various polystyrene copolymeric surfaces has revealed that vitronectin has a greater affinity for surface binding [43]. While all MNPs exhibited negative surface charge and relatively high levels of vitronectin adsorption, fibronectin was not observed. A similar trend in the adsorption of vitronectin and fibronectin has been found in β-CD-grafted MNPs [27].

α_1_-antitrypsin is a serine protease inhibitor that accounts for 95% of trypsin inhibitory capacity and has immunomodulatory properties, such as anti-inflammatory effects and T- and B-lymphocyte regulation [45,46]. All MNP types showed moderate absorption levels of α_1_-antitrypsin. Given the anti-inflammatory nature of α_1_-antitrypsin, this protein acts as an immune system regulator in connection to nanoparticles, where the presence of α_1_-antitrypsin in the protein corona of nanoparticles has been shown to decrease macrophage recall to the nanoparticle site [47]. Previous studies on MNPs grafted with β-CD have shown slightly higher levels of α_1_-antitrypsin adsorption [27].

α_2_-macroglobulin inhibits a wide range of proteases and regulates proteases by enhancing their clearance from blood [48,49]. Very faint bands were observed for α_2_-macroglobulin on bare and α-MNPs, but none was detected on β- and γ-MNPs. This correlates with previous studies where α_2_-macroglobulin has not been detected adsorbed to β-CD-grafted MNPs [27].

### 2.9. Plasma Proteins Involved in Coagulation

Fibrinogen (340 kDa) is composed of three polypeptide chains (Aα—68 kDa, Bβ—56 kDa, and γ—48 kDa). It is a substrate for Factor XIIIa and thrombin for clot formation, where cleavage fragments may appear for MW < 48 kDa [34,50,51]. The adsorption level of all fibrinogen fragments remained relatively low and constant across all MNP systems. Fibrinogen cleavage fragments (<48 kDa) were moderate (4 scale) for modified MNPs, while bare MNP showed a very low quantity of cleavage fragments (1 scale). That said, fibrinogen adsorption alone can induce platelet adhesion and thrombosis. The relatively low levels of adsorbed fibrinogen are consistent with previous findings, where the β-CD coating has been used to reduce fibrinogen adsorption to polyethylene- and polypropylene-based biomaterials, leading to improved hemocompatibility [52]. While previous studies have demonstrated that cleavage fragments exhibit low quantity in the presence of MNPs grafted with β-CD, these MNPs exhibited relatively high levels of adoption for all three fibrinogen polypeptide chains [27].

Prothrombin is transformed to form the active enzyme thrombin that is critical to the coagulation cascade [53]. Very low and consistent levels of prothrombin were found for all MNP systems. The active site of thrombin is a substrate for antithrombin (53 kDa), a serine protease inhibitor that acts as an endogenous anticoagulant. Antithrombin complexes with thrombin and other coagulation factors to inhibit coagulation [54]. Bare MNPs were found to adsorb relatively high levels of antithrombin, whereas CD coating resulted in a step decrease in the adsorption of this protein. β-CD-grafted MNPs have shown similar adsorption levels for prothrombin and antithrombin, with the intensity of antithrombin remaining consistent between bare and modified MNPs [27].

The contact pathway of coagulation involves factors XII and XI, plasma prekallikrein, and high molecular weight kininogen, which serves as a non-enzymatic cofactor [55]. Factor XI has been previously found in the protein corona of glucose-coated iron oxide particles and is known to play a role in nanoparticle clearance [56]. Factor XI is absorbed at high and relatively high levels by all MNP types. A moderate amount of Factor XII was absorbed to all MNP systems at similar levels, despite previous work showing that increasing particle size from ~10 nm to ~75 nm increased of Factor XII adsorption to silica nanoparticles [57]. Kininogen, known for its involvement in initiating the contact activation pathway, was not found in any of the formulations. The intensity values for factors XII and XI, and kininogen, correlated with previous findings on MNPs grafted with β-CD [27].

Prekallikrein (85 kDa) cleaves high-molecular-weight kininogen [58] and exhibited consistently low adsorption levels across all MNP types. The activated form of prekallikrein and kallikrein (50 kDa) displayed bands with slightly higher intensity than prekallikrein for all MNP systems. The reduction in the intensity of prekallikrein immunoblot bands is believed to be related to the activation of the contact system, with the formation of kallikrein complexes with α_2_-macroglobulin and a C1 inhibitor [59,60]. Prekallikrein has been found at very low adsorption levels in β-CD-grafted MNPs, with a substantial increase in the intensity value of kallikrein (50 kDa) [27].

Plasminogen (91 kDa) is the zymogen of plasmin, the primary protease responsible for fibrinolysis [61]. β- and γ-MNPs showed the decreased adsorption of plasminogen compared to bare and α-MNPs, where they exhibited relatively high adsorption levels for this protein. Plasminogen adsorption could indicate fibrinolytic activity [62]. While the adsorption levels have been reported as relatively high, MNPs grafted with β-CD have previously demonstrated a one-step decrease in plasminogen adsorption compared to bare MNPs [27].

Protein S regulates coagulation and acts as a cofactor for activated protein C [63]. Additionally, Protein S demonstrates anticoagulant functions independently of activated protein C; it directly inhibits intrinsic tenase and prothrombinase complexes [64]. None of the MNPs exhibited fibronectin, protein S, or protein C adsorption, indicating a restricted clot formation and fibrinolytic reaction [34]. These findings correlate with previous studies, where β-CD-grafted MNPs have not exhibited the adsorption of the aforementioned proteins [27].

### 2.10. Whole Blood Hemocompatibility and Platelet Function

**Figure 8:**
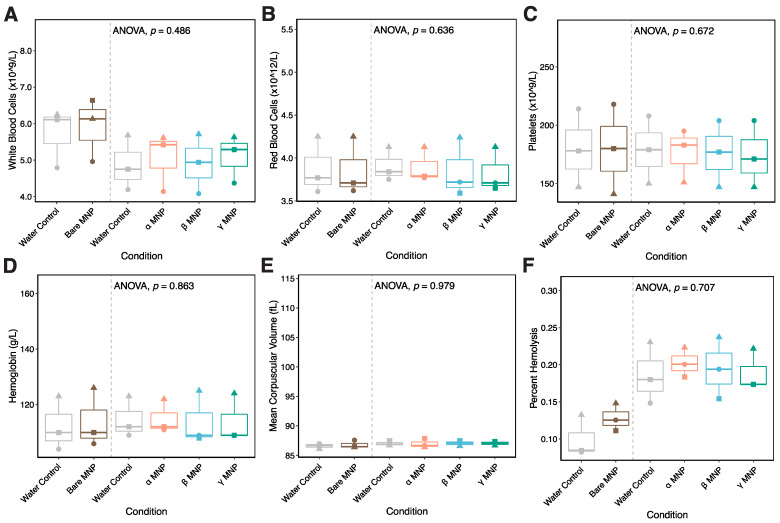
CD-coated MNP exposure does not impact complete blood counts or hemolysis. Whole blood from N = 3 healthy donors was incubated with 0.18 mg/mL of each MNP formulation. Sysmex XN-550 hematology analyzer results for (**A**) white blood cells, (**B**) red blood cells (**C**) platelets, (**D**) hemoglobin, and (**E**) mean corpuscular volume of red cells are shown. Hemolysis was assayed in MNP-depleted plasma via the Harboe method (**F**). Data on the left side of the dotted line is from a separate set of experiments, previously published by our group [27], and shapes reflect biological replicates. Results were compared across groups with repeated measures ANOVA to compare differences within biological replicates across groups, and paired t-tests were used for pairwise comparisons to the water control. Comparisons not shown were not statistically significant.

To further investigate the hemocompatibility of these MNPs, experiments were conducted after 1h incubations at 37 °C of MNPs with whole blood. Complete blood counts were performed (Figure 8A–E) to probe cellular changes in response to MNP exposure. As expected, there is clear variability in the measures across donors, but MNPs do not appear to impact white blood cell (Figure 8A), red blood cell (Figure 8B), or platelet (Figure 8C) counts compared to the control—indicating integrity was not compromised. There was also no effect of surface coating on hemoglobin (Figure 8D) or the mean corpuscular volume of the red blood cells (Figure 8E).

Hemolysis was assessed in plasma through the quantification of free hemoglobin via spectrophotometry using the Harboe method [65]. While all CD coatings showed similar percent hemolysis to the water control, it was evident that hemolysis was higher in this set of experiments compared with our previously published data (shown on the left side of the dashed line). This is likely a direct result of the lower concentration of the stock suspensions of MNPs (8 mg/mL) used in these experiments compared with the previous dataset (10 mg/mL), requiring more volume to be added to reach a final concentration of 0.18 mg/mL. As the MNPs were suspended in water, it follows that decreased osmotic pressure in this set of experiments would yield increased hemolysis, which is in line with the increased hemolysis in the control. Overall, the data does not suggest hemolysis induced by the CD-modified MNPs.

**Figure 9:**
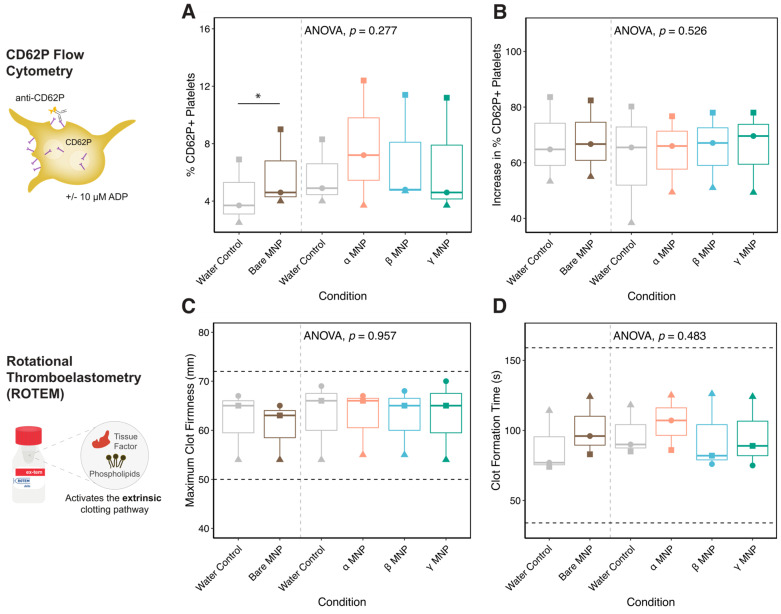
Platelet function is not impaired by CD-coated MNP exposure. Whole blood from n = 3 healthy donors was incubated with 0.18 mg/mL of each type of MNP. (**A**) Baseline platelet activation reflected by the surface expression of CD62P detected by flow cytometry. Percentage of CD62P+ platelets displayed. (**B**) Platelet degranulation in response to 10 μM of ADP, reflected by the surface expression of CD62P, detected by flow cytometry. Baseline activation was subtracted from the % of CD62P+ platelets to yield an increase in the degranulated platelets, as a measure of the platelet response. (**C**) Platelet function in coagulation reflected by the ROTEM maximum clot firmness and (**D**) clot formation time. Horizontal dashed lines indicate normal ranges as per the manufacturer’s information, and shapes reflect biological replicates. Data on the left side of the dotted line is from a separate set of experiments, previously published by our group [27]. Results were compared across groups with repeated measures ANOVA to compare differences within biological replicates across groups, and paired t-tests were used for pairwise comparisons to the water control (* *p* < 0.05). Comparisons not shown were not statistically significant.

As platelet function is not entirely captured by changes in numbers on a complete blood count, the impact of modified MNP exposure on platelets was further assessed through flow cytometry (Figure 9A,B) and platelet-dependent rotational thromboelastometry outcomes (Figure 9C,D). Baseline platelet activation reflected by the percent of platelets with surface CD62P (Figure 9A) was not statistically significantly different from the control condition for any of the modified MNPs, although one donor (squares) had increased platelet activation with all three CDs, and α-MNP induced an increased activation in two of the three donors. Even so, the increases were marginal and likely not clinically significant. Bare MNPs consistently increased platelet activation across all three donors, but also to a marginal extent. The lack of consistent increase in platelet activation with exposure to modified MNPs indicated that the polymer coating may enhance compatibility with platelets when compared with bare MNPs. The platelet responsiveness to ADP (Figure 9B) was not significantly different in any of the modified MNPs, indicating retained function and responsiveness.

Platelet-dependent outcomes in whole blood rotational thromboelastometry were also not significantly impacted by exposure to modified MNPs. Maximum clot firmness, which is a product of fibrin polymerization and platelet stabilization of the clot, was not affected by MNP exposure (Figure 9C). Clot formation time—the time from clot initiation to a firmness of 20 mm—was also not impacted by treatment with any of the MNPs (Figure 9D).

Taken together, the hemocompatibility experiments in whole blood suggest that all of the CD coatings tested are compatible with whole blood and merit further exploration for their ability to clear and mitigate the adverse effects of uremic toxins in the blood of CKD patients on hemodialysis. Importantly, many of these experiments were conducted without the removal of the MNPs from the whole blood/plasma. Given that the modified MNP adsorbs several critical proteins for coagulation and the immune response (Table 3), further evaluation is required to determine whether hemostasis is maintained following their removal, as would occur in the clinic. In addition to their target effect of uremic toxin binding, they may also reduce the levels of key proteins via adsorption and subsequent removal from circulation, but the extent of this reduction and whether it would be a clinically relevant decrease remains unknown.

## 3. Conclusions

This hemocompatibility study of α-, β-, and γ-MNPs as potential adsorbent biomaterials for uremic toxin clearance revealed several key findings. The surface coating of iron oxide MNPs with CDs improved particle characteristics for biomedical applications, resulting in smaller cluster sizes and a more uniform size distribution. Circular dichroic analysis revealed that the modified MNPs had minimal effects on HSA and α-lactalbumin. Lysozyme exhibited a subtle alteration, demonstrating a 2.1–2.3% decrease in α-helix and a 0.1–0.4% increase in β-sheet content. Notably, γ-CD-coated MNPs induced the most significant changes, leading to a 0.8% reduction in the helix content of α-lactalbumin and a 0.6% increase in the random coil content of HSA. A consistent trend in quenching in tryptophan fluorescence was observed, with a gradual increasing of the concentration of MNPs in the protein solution, aiding in the determination of binding sites and affinity. β-MNPs exhibited the lowest, while α-MNPs showed the highest number of binding sites. However, the binding affinity towards HSA remained relatively consistent across different MNP systems.

A total protein assay was performed to assess the quantity of protein adsorbed to each type of MNP, revealing that bare MNPs exhibited the highest protein absorption per unit of surface area. Additionally, an immunoblot analysis of MNP-associated proteins was conducted, probing 21 plasma proteins. Among these, 14 proteins were identified as being associated with these MNP systems. Notably, the immunoblot results indicated variations in the adsorption levels of C3, fibrinogen, antithrombin, Factor XI, and plasminogen across different MNP systems. The absence of fibronectin and proteins S and C within the protein corona of MNPs indicates a potential limitation in the fibrinolytic response. Despite the observed interaction between prekallikrein and MNPs, the absence of kininogen provides valuable insights that may contribute to refining blood-contacting adsorbents. When evaluating the hemocompatibility of whole blood across three biological replicates, no significant MNP-related effects were observed on the erythrocyte or leukocyte counts. The CD modification did not show any negative impact on platelets, as evidenced by the absence of detectable increases in platelet activation and retained function as assessed by rotational thromboelastometry. Overall, these findings contribute to the understanding of the hemocompatibility profile of CD-coated MNPs, providing essential insights for their potential application as absorbent biomaterials in the clearance of uremic toxins from blood.

## 4. Materials and Methods

### 4.1. Materials

Human plasma: Platelet-poor human plasma was obtained through the Blood4Research program from Canadian Blood Services (REB: 2022.021) as approved by the research ethics board of the University of Alberta.

Chemicals for synthesis: FeCl_2_·4H_2_O and FeCl_3_·6H_2_O (>98%, Acros Organics, Geel, Belgium). Ammonium hydroxide solution (25%) was purchased from Sigma Aldrich, with β-Cyclodextrin (≥97%, Sigma Aldrich, St. Louis, MO, USA), α-Cyclodextrin (≥98%, Sigma Aldrich), γ-Cyclodextrin (≥98%, Sigma Aldrich), Sodium Citrate (Fisher Scientific, Waltham, MA, USA).

Chemicals for experiments: Sodium phosphate monobasic monohydrate, Sodium phosphate dibasic heptahydrate, and PBS tablet were purchased from Fisher Scientific. Sodium dodecyl sulfate (SDS) and polyvinylidene fluoride (PVDF) membranes were from Bio-Rad, Hercules, CA, USA. Total protein assays were conducted using Pierce™ BCA Protein Assay Kit (Thermo Fisher Scientific Inc., Waltham, MA, USA). The TMB-stabilized substrate was purchased from Promega (Madison, WI, USA). For the complete list of primary and secondary antibodies, see Supplementary Table S1. All chemicals were used without further purification.

### 4.2. Methods

#### 4.2.1. Synthesis of CD-Coated MNPs

CD-MNPs synthesis was carried out as previously detailed [20,21]. Milli-Q water degassed (1 h, at room temperature) and purged (UHP N_2_, 20 min), and 25 mL of 100 μg of CD along Fe^3+^ and Fe^2+^ ions (2:1 molar ratio), was introduced in a nitrogen-inlet-equipped three-necked, round-bottom glass flask. The mixture was stirred (400 rpm, 20 min) and 1.5 mL of ammonium hydroxide solution was added dropwise at a consistent rate while stirring at 900 rpm. The resulting nanoparticle suspension was gently stirred overnight to facilitate the volatilization of excess ammonia. The nanoparticle precipitates underwent a thorough rinsing process with Milli-Q water. The nanoparticle suspension was stirred overnight with sodium citrate (500 mg/mL) solution. The resultant product then underwent another Milli-Q water wash, and the nanoparticle suspension in water was stored at 4 °C for subsequent characterization.

#### 4.2.2. Characterization of MNPs

Thermogravimetric analysis (TGA) was conducted (TGAQ50, TA Instruments, New Castle, DE, USA) under a 60 mL/min nitrogen gas flow and temperature range of 25 to 950 °C with a 10 °C/min ramp. TEM images were acquired (Philips-FEI Morgagni-268 transmission electron microscope, Hillsboro, OR, USA) at an acceleration voltage of 80 kV. The surface zeta potential and hydrodynamic size of CD-modified MNPs were evaluated using (Zetasizer Nano ZS, Malvern Instruments, Malvern, UK) as outlined previously [20]. Briefly, MNP suspensions (8 μg/mL in Milli-Q water) underwent a 30-s sonication and were promptly characterized.

#### 4.2.3. Fluorescence Spectroscopy

The accessibility of tryptophan residues in HSA structure was tested via MNP quenching studies following a previously established procedure [20]. In summary, HSA (330 µg/mL) in a 10 mM PB solution at pH 7.4 was gradually mixed with various MNP formulations (1 mg/mL) and the fluorescence spectra captured (FlexStation 3 multimode plate reader, Molecular Devices). Emission fluorescence intensity was recorded (300 to 500 nm) using an excitation wavelength of 280 nm. The binding affinity constant (K_b_) and the number of binding sites (n) were determined using established methodologies (Equation (1)) [20,66,67].

(F_0_ − F)/(F_0_ − F_s_) = [(S)/K_d_]^n^ (1)

where F_0_ is the relative fluorescence intensity (F) of the protein solution alone, F_s_ is the relative fluorescence intensity of protein saturated with MNPs, and [S] is the concentration of MNPs. n is the number of binding sites and was determined from the slope of the plot, log [(F_0_ − F)/(F − F_s_)] vs. log [S]. The log [S] at log [(F_0_ − F)/(F − F_s_)] = 0 determines the logarithm of the dissociation constant (K_d_), where K_b_ is the reciprocal of K_d_.

#### 4.2.4. SDS-PAGE and Immunoblot

Upon the arrival of pooled platelet-poor plasma, the plasma samples were aliquoted and stored at −80 °C until needed. The incubation of MNPs with platelet-poor plasma was carried out following established protocols [34]. Plasma was combined with MNPs (0.18 mg/mL) at 37 °C for 2 h, centrifugated (20,000 × *g* for 10 min), supernatant removed, and MNP pellet washed twice (1 mL, 1xPBS) to eliminate loosely bound proteins. Final pellet was resuspended in 100 μL of 10% SDS in PBS and incubated at 50 °C for 2 h to elute adsorbed proteins. Eluted protein concentrations were determined using a detergent-compatible Pierce™ BCA protein assay.

SDS-PAGE and immunoblot techniques were employed for sample analysis, following a previously documented protocol [34,38]. An equal amount of each protein sample (30 μg) was loaded onto SDS-PAGE gels.

Gels were transferred to polyvinylidene difluoride membranes, which were then cut into 23 strips. Two strips were used for colloidal gold staining, while 21 were utilized for protein characterization. Primary antibodies (1:1000 dilution) were applied and incubated for 1 h at room temperature. HRP-conjugated secondary antibodies with TMB substrate were used for visualization. All immunoblots underwent a consistent 5-min color development for intensity comparison.

#### 4.2.5. Circular Dichroism

Far-UV CD (Olis DSM 17 Circular Dichroism Spectrometer, Olis, Bogard, GA, USA) spectra of HSA, α-lactalbumin, and lysosome were obtained using protein concentrations of 1.25, 0.25, and 0.25 mg/mL for HSA, α-lactalbumin, and lysozyme, respectively. CD-MNPs were at a concentration of 0.25 mg/mL. Equal volumes of the protein and MNP solutions were mixed and incubated for 3 h at 37 °C before the test. The CD spectra were recorded in the range of 180 to 260 nm. The reported results represent the average values obtained from three independent repetitions. The raw data were further analyzed using CDNN 2.0 software to articulate the changes in secondary structure upon the addition of MNPs.

#### 4.2.6. Whole Blood Hemocompatibility Testing

For whole blood hemocompatibility testing, venous blood from N=3 healthy donors was collected into 2.7 mL BD vacutainers containing buffered sodium citrate (0.109 M, 3.2%). All donors provided informed consent, and this study was approved by the UBC Clinical Research Ethics Board (H22-00215). MNPs suspended in deionized water (or an equal volume of deionized water, as a control) were added to 2.7 mL of citrated whole blood at a final concentration of 0.18 mg/mL and incubated on a rocker for one hour at 37 °C. Hemocompatibility of bare MNPs was obtained in a separate but parallel set of experiments, as previously published [27], and shown on the graphs separated by a dashed line to indicate separate datasets.

Following incubation, complete blood counts were assessed using a Sysmex XN-550 hematology analyzer (Sysmex Corporation, Kobe, Japan). Coagulation was assessed in the treated citrated whole blood using rotational thromboelastometry (ROTEM; Instrumentation Laboratory, Bedford, MA, USA), where 300 μL of whole blood was added to the STAR-TEM and EXTEM reagents to re-calcify and activate the extrinsic coagulation pathway, respectively. Primary outcomes of interest included the clotting time (the period from the beginning of coagulation to the start of fibrin polymerization), the clot formation time (time from clot initiation to a firmness of 20 mm—reflects fibrin polymerization and stabilization with platelets), and the maximum clot firmness (mechanical strength of the clot—dependent on platelet function, fibrin polymerization, and Factor XIII activity).

Baseline platelet activation and responsiveness to a weak agonist were evaluated using flow cytometry on a BD FACSCanto II flow cytometer (BD Biosciences, San Jose, CA, USA). For baseline activation, 3 μL of whole blood was incubated with 5 μL of mouse anti-CD62P (IM1759U, Beckman Coulter, Brea, CA, USA) in 0.22 μm filtered PBS at a total volume of 50 μL. For responsiveness, 3 μL of whole blood was incubated with ADP (Chrono-Log, Havertown, PA, USA) at a final concentration of 10 μM and 5 μL of antibody in 0.22 μm filtered PBS, at a total volume of 50 μL. After a 30-min incubation at room temperature, samples were diluted with 0.75 mL of 0.22 μm filtered PBS before measurement. The location of the platelet population on the FSC/SSC plot was confirmed with a CD41 stain and gated for the remainder of the experiments. Gates for a positive CD62P signal in the platelet population were determined for each experiment based on an isotype control (IgG1; IM0670U, Beckman Coulter). Platelet responsiveness to ADP was calculated by subtracting baseline (unstimulated) % CD62P+ from the total % CD62P+ following stimulation with ADP.

The remaining whole blood was centrifuged at 3000 rpm for 10 min at 4 °C to obtain platelet-poor plasma (PPP). The PPP was spun again at 20,000 × *g* for 20 min at 4 °C to pellet the remaining microparticles before exposure to a magnet for 15 min at room temperature. Hemolysis was measured in PPP using the previously described Harboe method [65], and C3a levels reflecting complement activation were assessed in MNP-depleted plasma aliquots frozen at −70 °C using a commercial ELISA (#A031, Quidel, San Diego, CA, USA), following the kit instructions for plasma. All diluted samples were within the detection limits of the ELISA.

Graphing and statistical analysis for the hemocompatibility outcomes were performed in RStudio using R version 4.0.5, with the packages rstatix, dplyr, grid, and ggplot2. Due to the innate variability in whole blood characteristics across donors, hemocompatibility results were evaluated using a repeated measures ANOVA, with the donor as the identifier and treatment as the within-subject variable. ANOVAs were only run on the data collected in this set of experiments (water control, α-, β-, γ-MNP). Pairwise comparisons were performed using paired t-tests, where each MNP formulation was compared to the water control from the same experiment. P-values were not adjusted for multiple comparisons, as the goal was to identify any MNP formulations that showed indications of hemo-incompatibility with high sensitivity.

## Figures and Tables

**Table 1:** Summary of size and zeta potential measurements. DLS and zeta potential measurements, n = 3. TEM data represent n = 100.

MNP Type	Cluster Size via DLS (nm)	Polydispersity Index (PI)	Particle Diameter via TEM (nm)	Zeta Potential (mV)
Bare	301 ± 23	0.65 ± 0.22	11.36 ± 2.30	5.0 ± 0.2 ^a^
α-MNP	94.4 ± 0.7	0.14 ± 0.02	8.69 ± 1.81	−24 ± 1.9 ^b^
β-MNP	99.2 ± 0.7	0.18 ± 0.03	8.50 ± 1.51	−22 ± 0.6 ^c^
γ-MNP	90.1 ± 0.1	0.19 ± 0.01	7.69 ± 1.37	−22 ± 0.5 ^d^

^a^ is statistically different from ^b^, ^c^, and ^d^ as analyzed via one-way ANOVA (*p* < 0.001). Data represent mean ± 1 SD, n ≥ 3.

**Table 2:** Representative results for the effect of bare, α-, β-, and γ-MNP on the secondary structure of α-lactalbumin, human serum albumin, and lysozyme. The unit is in percentage (%) and data represents mean ± 1 SD, n = 3.

	MNP Type	Helix	β-Sheet	β-Turn	Random Coil
Lysozyme	Blank	32.7 ± 0.1	8.1 ± 0.0	18.1 ± 0.0	28.5 ± 0.2
	α-	30.6 ± 0.0	8.2 ± 0.2	18.1 ± 0.0	28.9 ± 0.0
	β-	30.6 ± 0.2	8.2 ± 0.2	18.1 ± 0.0	28.9 ± 0.0
	γ-	30.4 ± 0.1	8.5 ± 0.0	17.8 ± 0.1	30.2 ± 0.3
	Bare	32.7 ± 0.3	8.1 ± 0.1	18.1 ± 0.0	28.5 ± 0.1
Human serum albumin	Blank	70.9 ± 0.0	4.1 ± 0.2	12.6 ± 0.1	12.2 ± 0.0
	α-	71.2 ± 0.1	4.1 ± 0.3	12.5 ± 0.2	12.1 ± 0.1
	β-	71.0 ± 0.0	4.1 ± 0.2	12.5 ± 0.1	12.3 ± 0.0
	γ-	70.3 ± 0.0	4.3 ± 0.1	12.8 ± 0.0	12.8 ± 0.3
	Bare	71.4 ± 0.1	4.1 ± 0.0	12.5 ± 0.2	12.1 ± 0.1
α-Lactalbumin	Blank	32.3 ± 0.2	20.8 ± 0.1	18.0 ± 0.0	28.9 ± 0.1
	α-	32.6 ± 0.0	20.3 ± 0.0	17.9 ± 0.1	29.3 ± 0.2
	β-	32.6 ± 0.1	20.3 ± 0.2	17.9 ± 0.1	29.3 ± 0.1
	γ-	31.5 ± 0.2	21.1 ± 0.3	17.9 ± 0.1	29.4 ± 0.2
	Bare	32.3 ± 0.2	20.8 ± 0.1	18.0 ± 0.0	28.9 ± 0.0

**Table 3:** Heat map illustrating the relative intensities of immunoblot of plasma proteins absorbed to different MNP types. Intensity levels are indicated as follows: 0: no band, 1 to 3: very low, 4 to 5: relatively low, 6 to 7: moderate, 8 to 9: relatively high, 10 to 11: high, and 12 indicating maximum intensity on the grayscale.

			MNP Type				
Plasma Proteins		Fragment Size (kDa)	Bare	α-CD	β-CD	γ-CD	
	Albumin	66	9	9	8	9	0
							1 to 3
Immune response-related	C3						4 to 5
							6 to 7
	Whole C3	187	4	4	3	3	8 to 9
	α chain	115	6	5	3	3	10 to 11
	β chain	70	9	9	8	8	12
	Activation fragment	42	6	6	6	6	
	IgG						
	Heavy chain	55	6	6	6	6	
	Light chain	27	9	9	8	8	
	Transferrin	77	9	9	8	8	
	Vitronectin	54	8	9	9	9	
	α_1_ antitrypsin	54	6	7	7	7	
	α_2_ macroglobulin	163	1	1	0	0	
Coagulation-related	Fibrinogen						
	α chain	68	5	5	4	4	
	β chain	56	4	4	4	4	
	γ chain	48	4	5	4	4	
	Cleavage fragments	<48	1	4	4	4	
	Prothrombin	72	2	2	2	2	
	Antithrombin	53	8	7	7	7	
	Factor I	88	0	0	0	0	
	Factor XII	80	6	7	7	7	
	Factor XI	70	9	9	10	10	
	Prekallikrein	85	5	5	4	4	
		50	7	6	6	6	
	Plasminogen	91	9	9	7	6	
	Kininogen	120	0	0	0	0	
	Fibronectin	440	0	0	0	0	
	Protein C	62	0	0	0	0	
	Protein S	69	0	0	0	0	

## Data Availability

The findings of this study are supported by the data provided in both the article and Supplementary Materials.

## Supplementary Materials

The following supporting information can be downloaded at: https://www.mdpi.com/article/10.3390/ijms251910710/s1.
