# Supplementary material for: Characterization and Hemocompatibility of α, β, and γ Cyclodextrin-Modified Magnetic Nano-Adsorbents
Source: Int J Mol Sci. 2024 Oct 4;25(19):10710. doi: 10.3390/ijms251910710 (PMC11476827; doi:10.3390/ijms251910710)
Supplement: Supplementary file 1 [file ijms-25-10710-s001.zip › ijms-3226089-supplementary.pdf]

Support information for

Characterization and Hemocompatibility of α, β, and γ Cyclodextrin-Modified Magnetic Nano-Adsorbents

Mehdi Ghaffari Sharaf [1,†], Shuhui Li [1,†], Elyn M. Rowe [2], Dana V. Devine [2], Larry D. Unsworth [1,*]

[1] Department of Chemical and Materials Engineering, University of Alberta, Edmonton, AB T6G 1H9, Canada.

[2] Department of Pathology and Laboratory Medicine, University of British Columbia, Vancouver, BC V6T 1Z7, Canada.

[†] These authors contributed equally to this work.

Author correspondence: Department of Chemical and Materials Engineering, University of Alberta, Edmonton, AB, Canada T6G 1H9.

Email: lunswort@ualberta.ca (L.D. Unsworth).

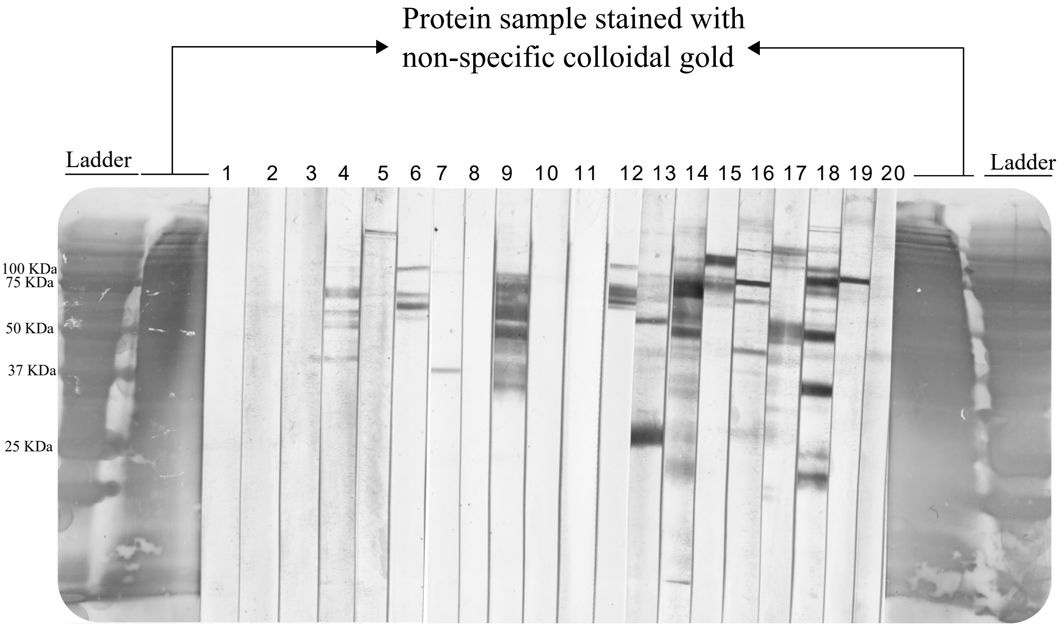

Figure S1. Representative reassembled western blot membrane of eluted plasma proteins.

1 Ani-kininogen (light)

2 Ani-kininogen (heavy)

3 Anti-factor I

4 Anti-fibrinogen α, β, and γ

5 Anti-fibronectin

6 Anti-Alpha$_1$ antitrypsin

7 Anti-prothrombin

8 Anti-protein C

9 Anti-vitronectin

10 Anti-protein S

11 Anti-Prekallikrein

12 Anti-antithrombin

13 Anti-IgG

14 Anti-human albumin

15 Anti-plasminogen

16 Anti-C3

17 Anti-factor XII

18 Anti-factor XI

19 Anti-transferrin

20 Anti-alpha$_2$ macroglobulin

Table S1. Primary antibodies against human plasma proteins used in immunoblot studies.

Anti-human plasma antibody	Host	Vendor
Albumin	Goat	OEM Concepts, Saco, ME, USA
Antithrombin	Sheep	Cedarlane Laboratories, Hornby, Ontario, Canada
Complement factor 3	Goat	Calbiochem, Gibbstown, NJ, USA
Factor I	Mouse	Invitrogen; Thermo Fisher Scientific Inc.
Factor XI	Goat	Cedarlane Laboratories, Hornby, Ontario, Canada
Factor XII	Goat	Cedarlane Laboratories, Hornby, Ontario, Canada
Fibrinogen	Rabbit	Calbiochem, Gibbstown, NJ, USA
Fibronectin	Rabbit	Cedarlane Laboratories, Hornby, Ontario, Canada
IgG	Goat	Sigma-Aldrich, St. Louis, MO, USA
Kininogen (heavy chain)	Mouse	US Biological, Swampscott, MA, USA
Kininogen (light chain)	Mouse	US Biological, Swampscott, MA, USA
Plasminogen	Goat	Cedarlane Laboratories, Hornby, Ontario, Canada
Prekallikrein	Sheep	Cedarlane Laboratories, Hornby, Ontario, Canada
Protein C	Sheep	Cedarlane Laboratories, Hornby, Ontario, Canada
Protein S	Sheep	Cedarlane Laboratories, Hornby, Ontario, Canada
Prothrombin	Sheep	Cedarlane Laboratories, Hornby, Ontario, Canada
Transferrin	Goat	Sigma-Aldrich, St. Louis, MO, USA
Vitronectin	Sheep	Cedarlane Laboratories, Hornby, Ontario, Canada
α1-Antitrypsin	Sheep	Cedarlane Laboratories, Hornby, Ontario, Canada
α2-Macroglobulin	Goat	Sigma-Aldrich, St. Louis, MO, USA
